# Publisher Correction: Quantification of bone marrow interstitial pH and calcium concentration by intravital ratiometric imaging

**DOI:** 10.1038/s41467-022-28925-1

**Published:** 2022-03-17

**Authors:** S-C. A. Yeh, J. Hou, J. W. Wu, S. Yu, Y. Zhang, K. D. Belfield, F. D. Camargo, C. P. Lin

**Affiliations:** 1 Advanced Microscopy Program, Center for Systems Biology and Wellman Center for Photomedicine, Massachusetts General Hospital, Harvard Medical School, Boston, MA 02114 USA; 2 Department of Chemistry and Environmental Science, New Jersey Institute of Technology, 323 Martin Luther King Jr. Blvd., Newark, NJ 07102 USA; 3 Stem Cell Program, Boston Children’s Hospital, Boston, MA USA; 4 Department of Stem Cell and Regenerative Biology, Harvard University, Cambridge, MA USA; 5 Harvard Stem Cell Institute, Harvard University, Cambridge, MA 02138 USA

**Keywords:** Ca2+ imaging, Haematopoietic stem cells, Stem-cell niche, Ca2+ imaging

Correction to: *Nature Communications* 10.1038/s41467-022-27973-x, published online 19 January 2022.

The original version of this Article contained an error in Fig. 1, Fig. 3, Fig. 4 in which the individual data points were inadvertently omitted. The correct version of Fig. 1 is:
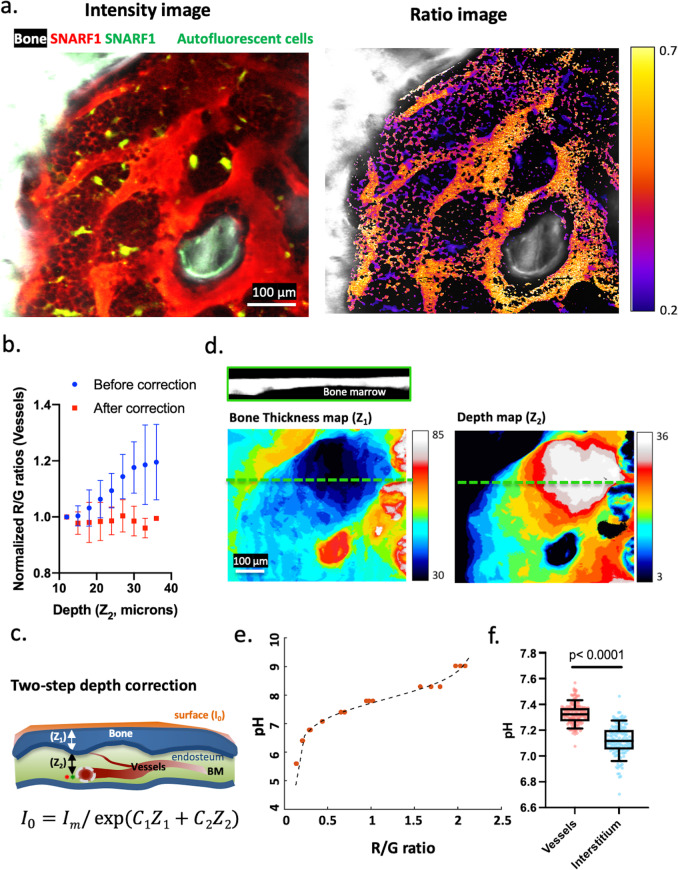


which replaces the previous incorrect version.
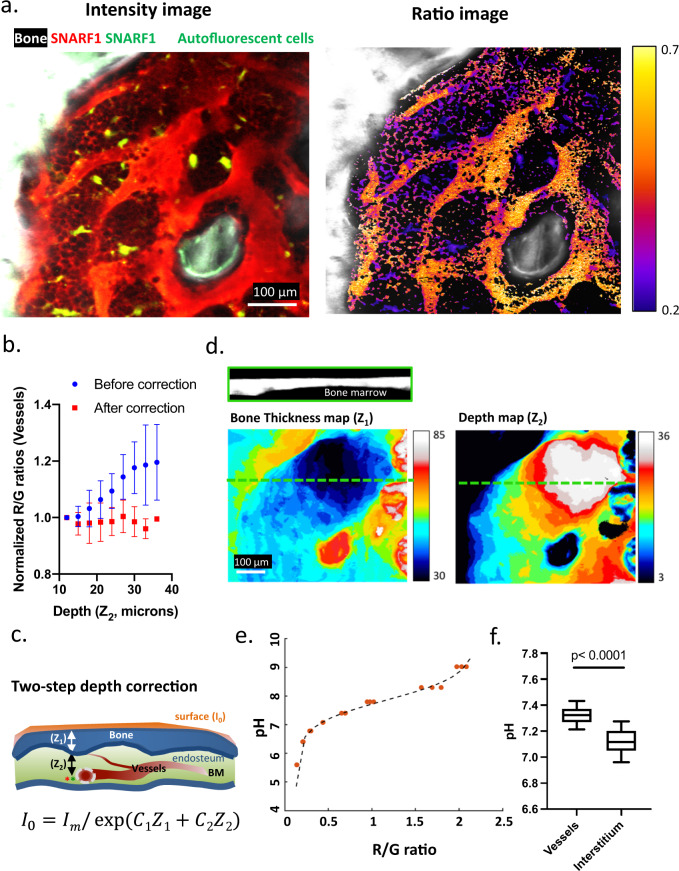


The correct version of Fig. 3 is:
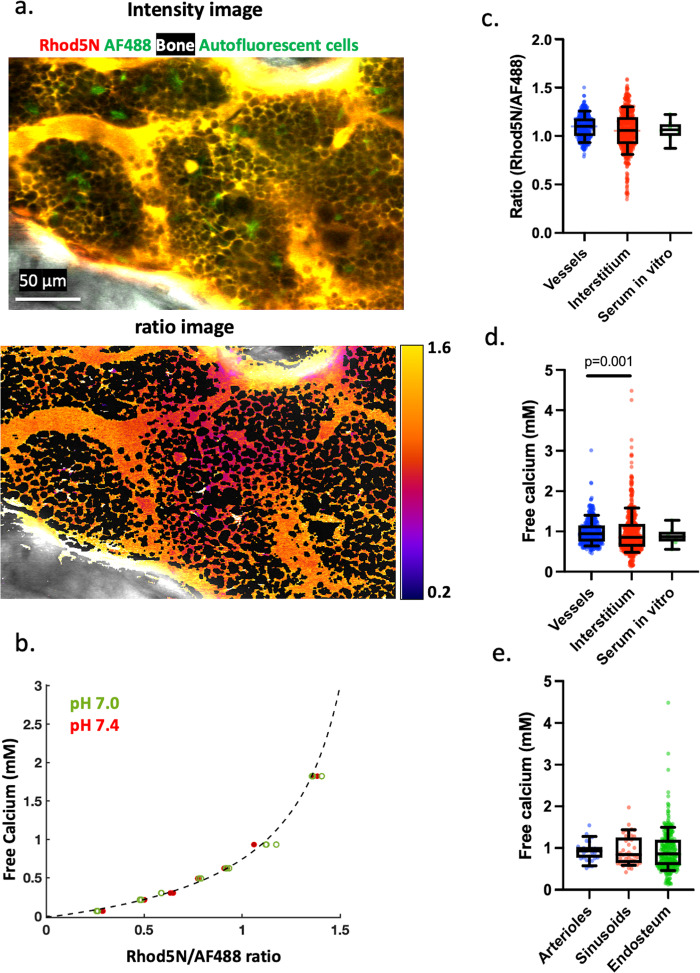


which replaces the previous incorrect version.
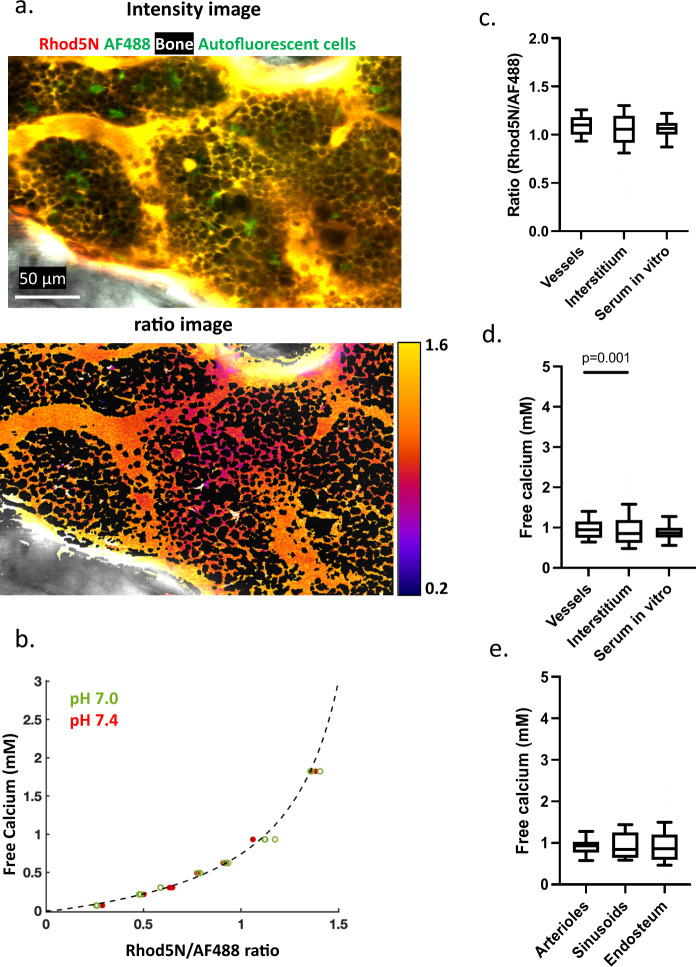


The correct version of Fig. 4 is:
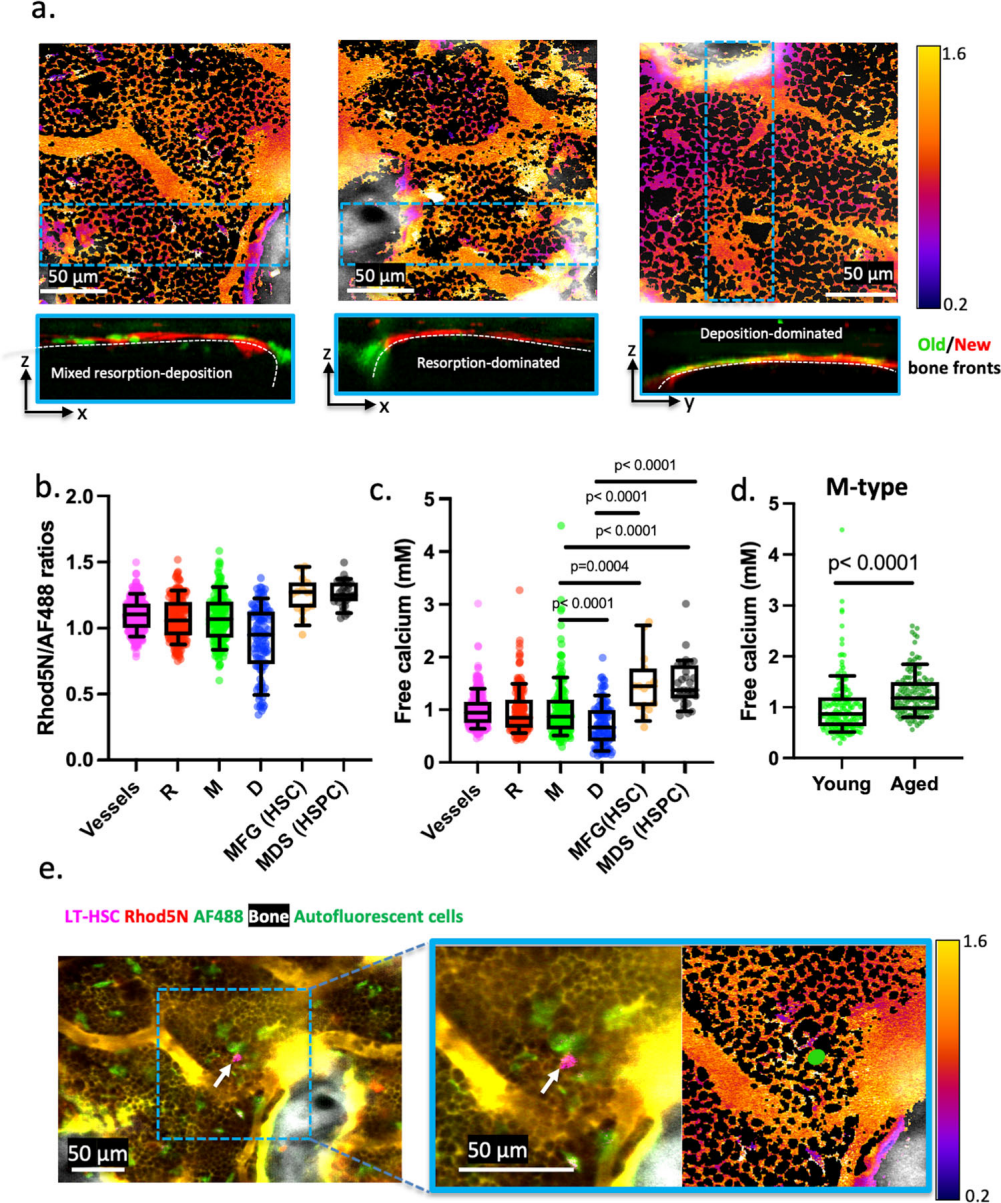


which replaces the previous incorrect version.
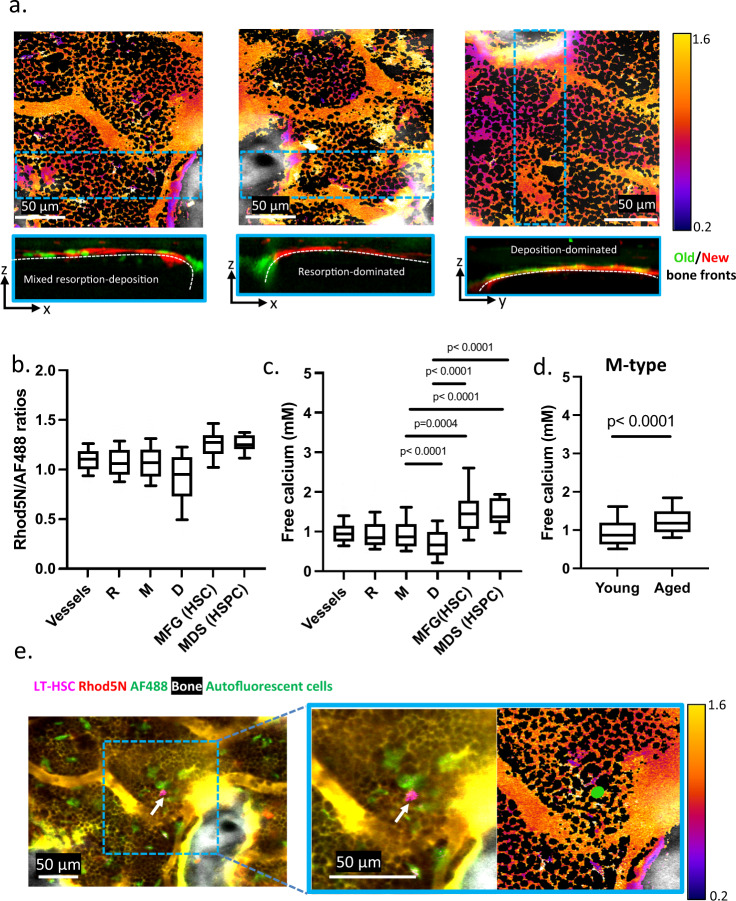


This has been corrected in both the PDF and HTML versions of the Article.

