# Supplementary material for: Quantification of bone marrow interstitial pH and calcium concentration by intravital ratiometric imaging
Source: Nat Commun. 2022 Jan 19;13:393. doi: 10.1038/s41467-022-27973-x (PMC8770570; doi:10.1038/s41467-022-27973-x)
Supplement: Supplementary file 1 — Supplementary Information [file 41467_2022_27973_MOESM1_ESM.pdf]

SUPPLEMENTARY INFORMATION

Figures

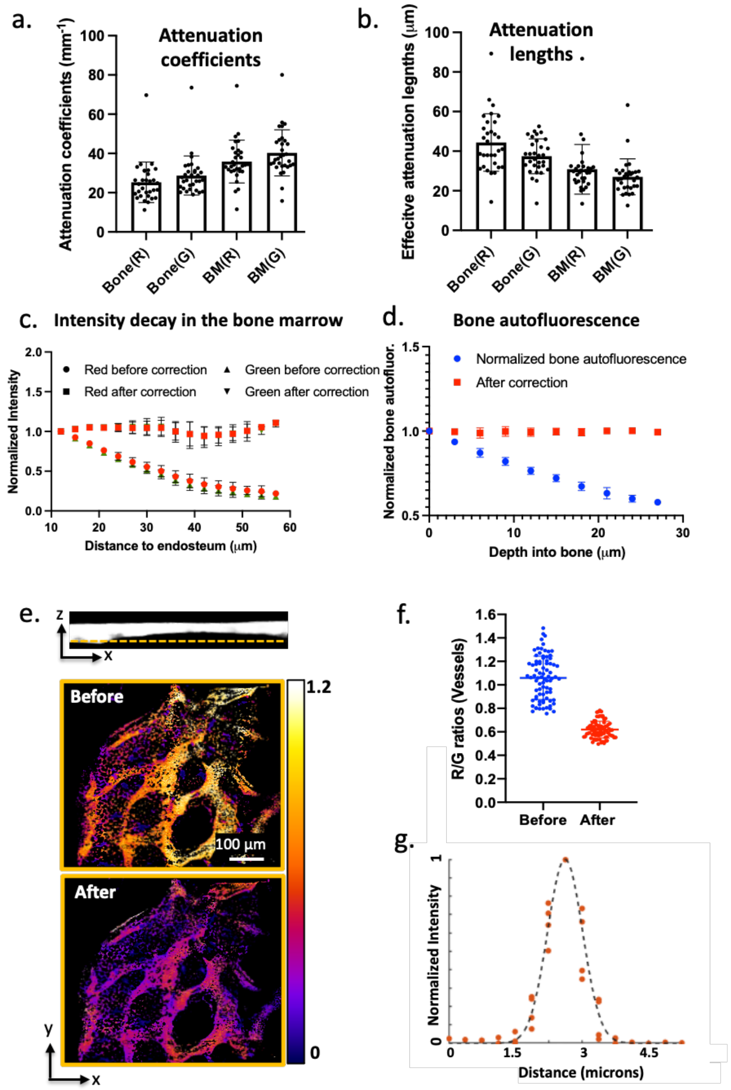

Supplementary Figure 1. Bone thickness and depth correction for SNARF-1. (A-B) The attenuation coefficients of red and green channels in bone (C_{1R}-C_{1G}) and in BM (C_{2R}-C_{2G}) were determined from the two-step depth correction algorithm as described in the Methods section. The corresponding effective attenuation lengths were determined by ($1/C_i$) (n=31 field of views, Mean ± s.d) **(C)** Fluorescence attenuation of Rhod-5N and AF488 in the BM vasculature. For each BM cavity, fluorescence at each depth was retrieved by averaging the intensity values from the segmented vascular regions located at a given distance to endosteum. (n= 6 BM cavities. Mean ± s.d). **(D)** Using the bone autofluorescence signal to derive the attenuation coefficient for the green channel. (n= 6 BM cavities. Mean ± s.d). **(E-F)** Ratio images from a single z-plane (orange dashed line) of the BM cavity. Ratiometric analyses without the two-step depth correction yielded divergent intravascular ratio, with increased R/G ratios observed at locations further from endosteum. Depth corrections minimized variation of intravascular ratios and recovered consistent pH in circulation. **(G)** Lateral resolution of the imaging system measured by drawing a line intensity profile across the interstitial space.

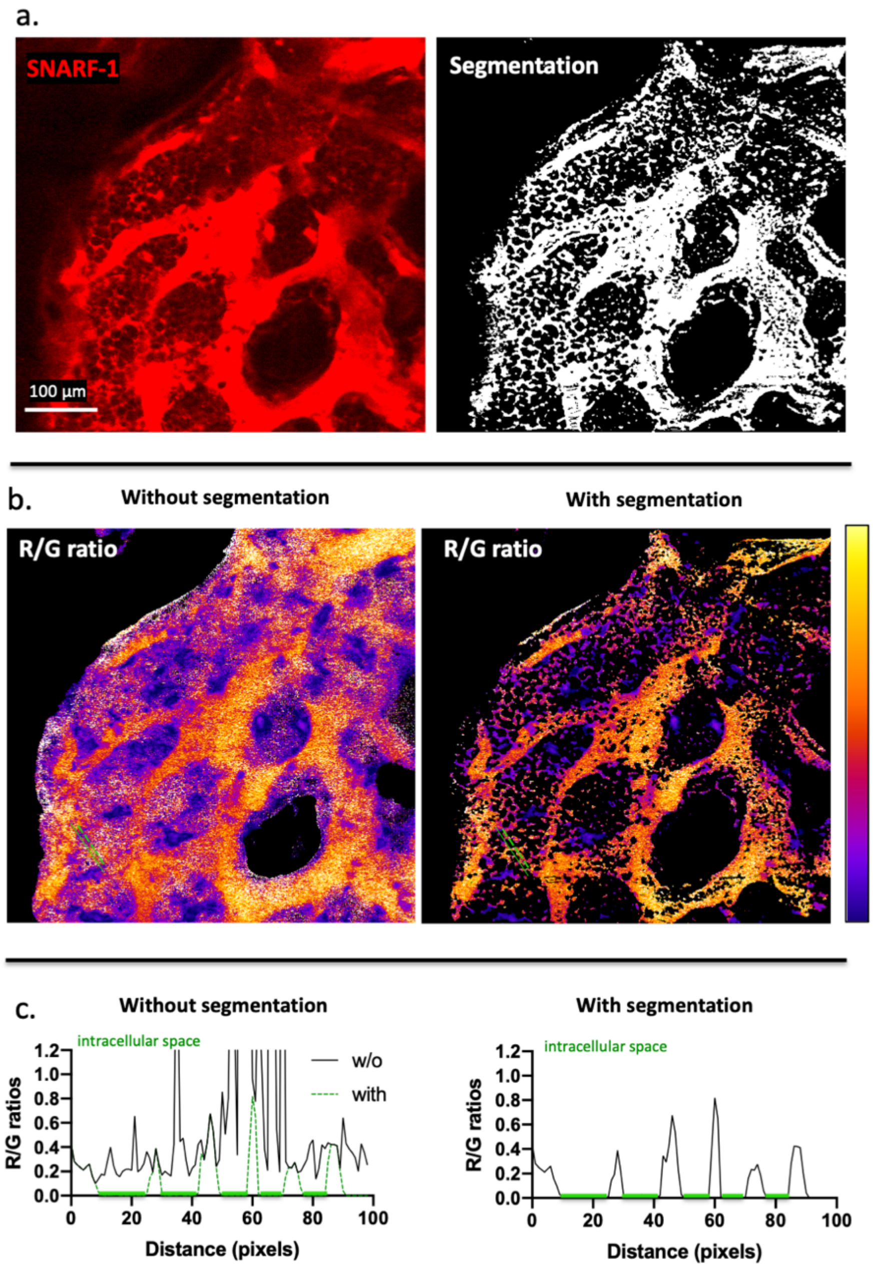

Supplementary Figure 2. Delineation of intravascular and interstitial space by image segmentation. (A) A representative image of BM labeled with SNARF-1 dextran. Image segmentation was based on the red channel and is a critical step to further delineate the vasculature and interstitium while excluding background noise and intracellular regions not labeled by the cell-impermeable dye. n= 10 BM cavities, N= 2 mice **(B)** Ratiometric analyses based on images without or with segmentation. White (infinity) pixels in the non-segmented images were due to zeros in the green channel (denominator in the ratio) after background subtraction. n= 10 BM cavities, N= 2 mice **(C)** Ratios changed across the green dashed line in **(B)** showing well-defined interstitial signals from the segmented image, whereas the signal is obscured by the noise (including infinity pixels) in the non-segmented image.

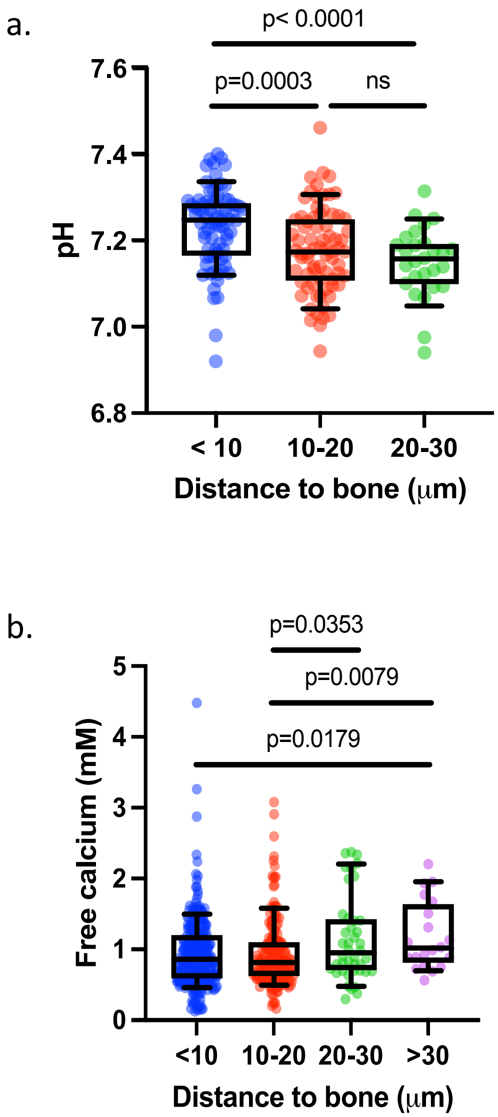

Supplementary Figure 3. pH and Calcium distributions with respect to the distance to bone. (A) Measured interstitial pH (N=3 animals, n=10 BM cavities, p<0.0003 and p<0.0001 between the group < 10 μm vs. > 10 μm and > 20 μm, respectively) and **(B)** $[Ca^{2+}]_e$ (N=10 animals. n=25 BM cavities) with respect to the distance to the endosteal surface (p<0.0179 between the group < 10 μm vs. > 30 μm; p<0.0353 and 0.0079 between the group 10-20 μm vs. 20-30 μm and > 30 μm, respectively). Box and whiskers represent the median, 25 and 75 percentiles, and the 10-90% data range. Two-sided Mann-Whitney test. Each data point represents an average ratio from the manually selected sub-ROI (~ 3-cell radius).

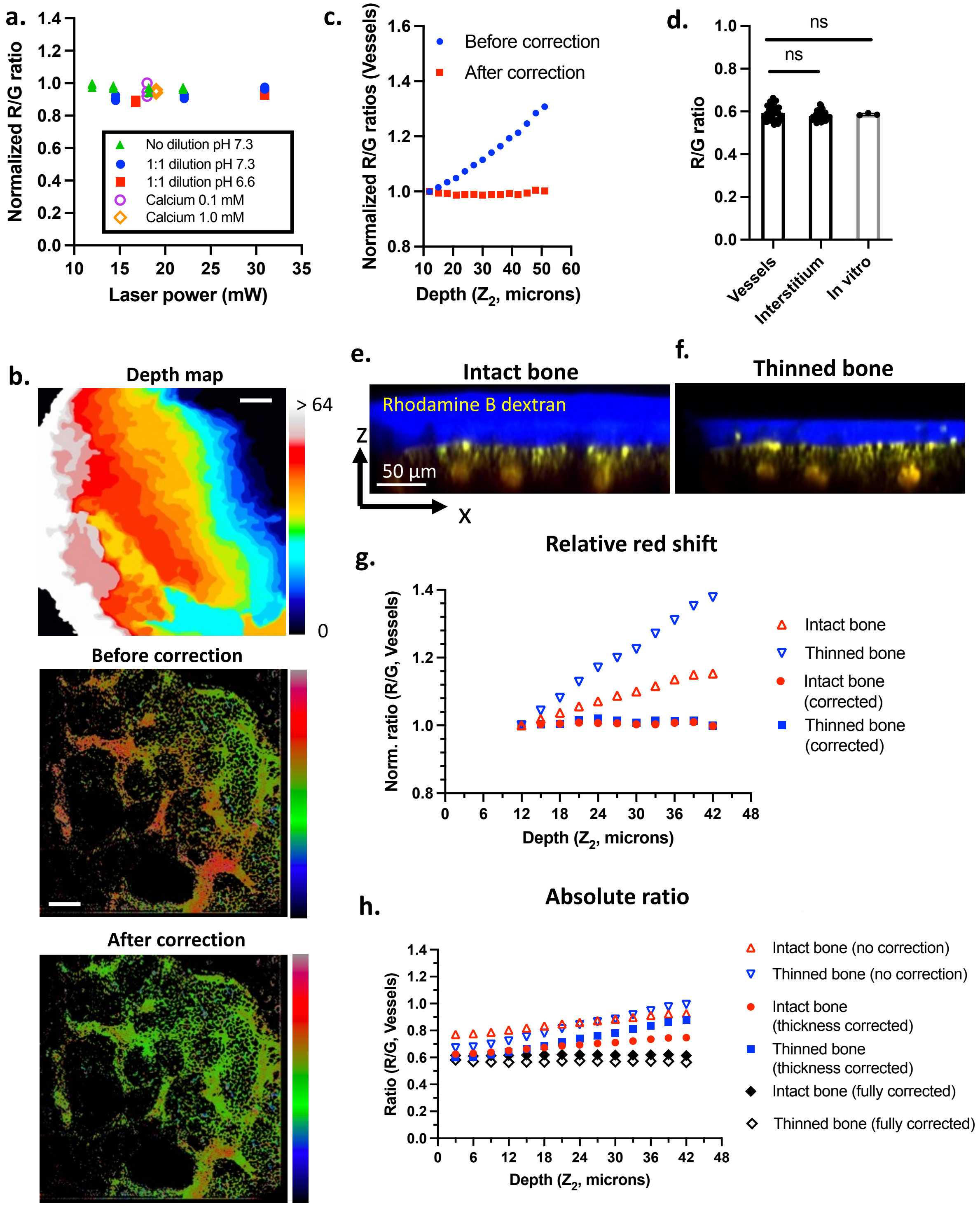

Supplementary Figure 4. Validation of the two-step depth correction method using Rhodamine-B dextran. (A) Rhodamine-B dextran is independent of the laser power, dye concentration, [Ca^{2+}], or pH in the physiologic range. **(B)** A depth map shows varying distances to endosteum from a single z plane (top). Corresponding ratio images at the same image plane as the depth map shows an increased R/G ratio at the deeper regions before correction (middle) and uniform R/G ratio after correction (bottom). **(C)** The normalized R/G ratios obtained from

vessels located at various distances to endosteum (depths) confirmed the convergence of intravascular ratio after depth correction. **(D)** The recovered intravascular R/G ratio (mean = 0.59 ± 0.034) is consistent with the in vitro measurements (ratio = 0.59 ± 0.005). No significant difference was observed between vessels and interstitium (ratio = 0.58 ± 0.021) (n = 30 and 31 sub-ROIs from vessels and interstitium, respectively. N = 1 mouse). Two-sided Mann–Whitney test. Mean ± s.d. **(E-F)** Cross-sectional images of calvarial bone and BM before and after laser bone thinning. (N = 1 mouse) **(G)** Intact bone and thinned bone exhibited a different extent of red shift and were recovered after the two-step depth correction. **(H)** The absolute R/G ratios plotted as a function of depth before correction, after bone thickness correction, or after completing the two-step depth correction. After bone thickness correction, the ratios at the endosteum from both intact and thinned were recovered close to the measured values in vitro. After both thickness and depth corrections, the ratios at all depths were recovered and consistent with the in vitro measurements.

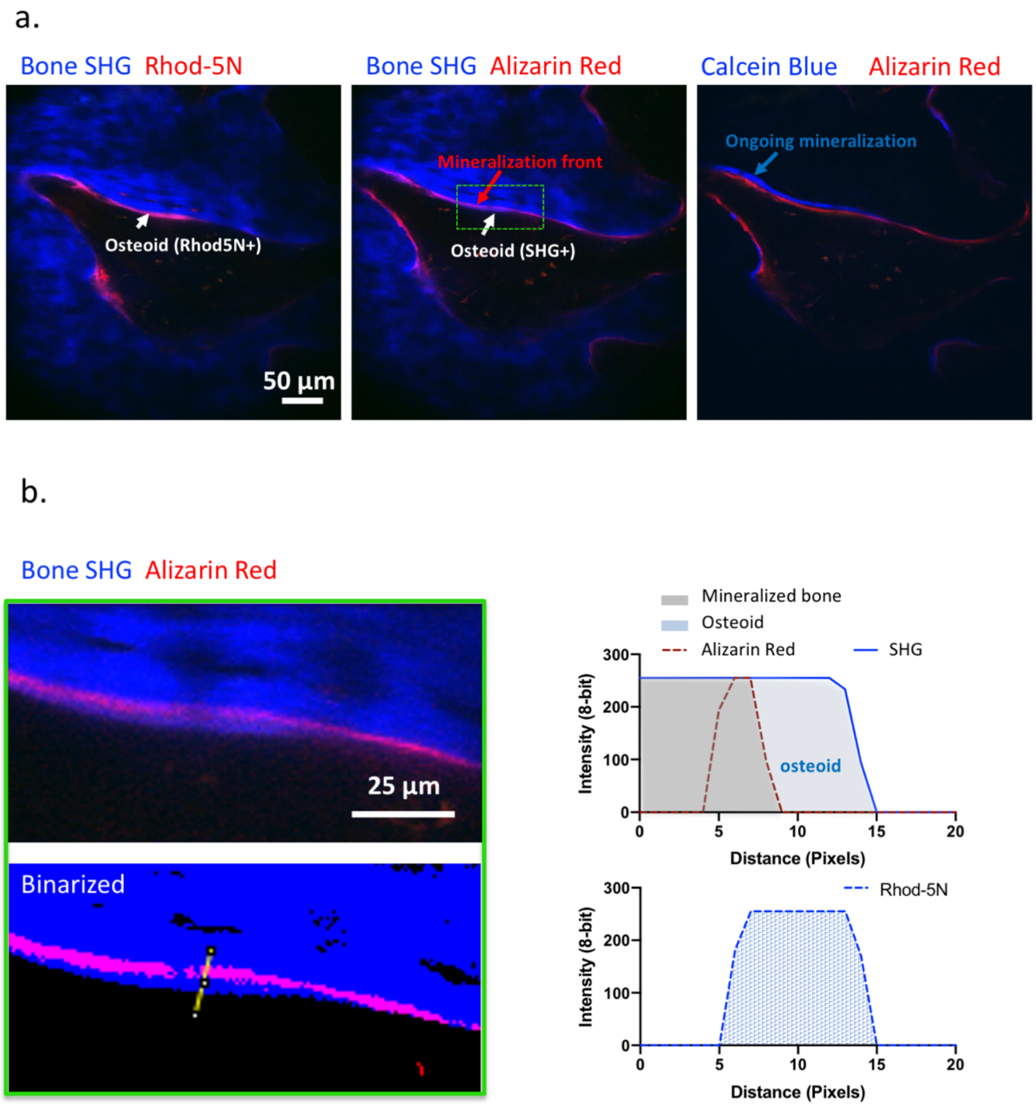

Supplementary Figure 5. Osteoids are strongly labeled by Rhod-5N. (A) Representative images showed a region with collagen structures (SHG) labeled by Rhod-5N, but not by Alizarin Red, indicating that the non-mineralized bone matrix with strong Rhod-5N signal correspond to osteoid, as Alizarin Red only binds to newly mineralized bone fronts. Sequential administration of bone front staining (Calcein Blue and Alizarin Red) further revealed that osteoids overlay regions with ongoing bone deposition activities. **(B)** A zoomed region (green dashed box in (A)), and the corresponding line intensity profiles based on the binarized image (yellow solid line). Line intensity profiles confirmed the non-mineralized collagen matrix (osteoid) that yields SHG signals, lacks Alizarin Red, and labeled by Rhod-5N. (n= 25 BM cavities, N= 10 mice).

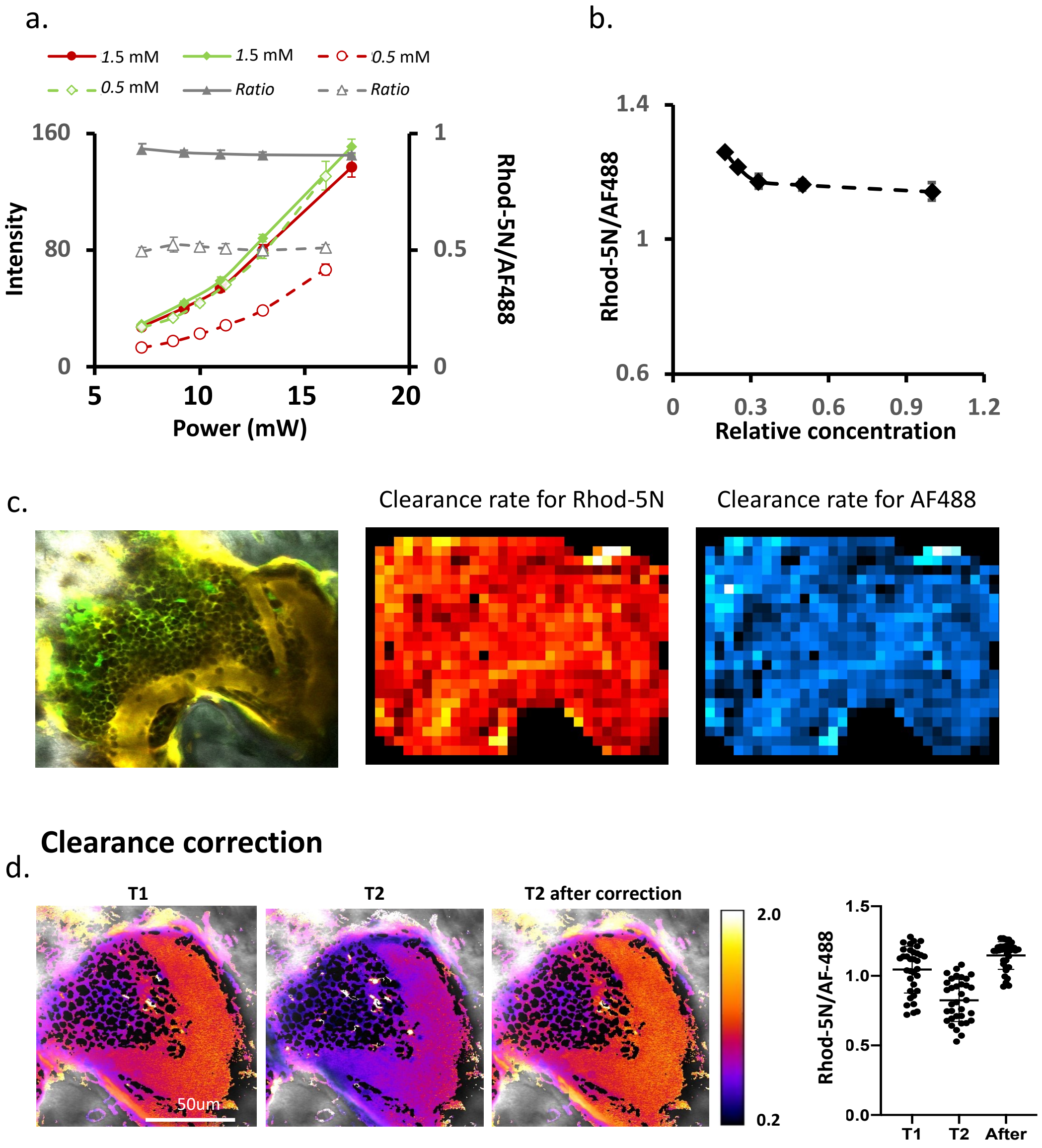

Supplementary Figure 6. In vitro calibration and clearance correction of ratiometric calcium measurements. (A) Power dependence of Rhod-5N and AF488 fluorescence dye pair. The fluorescence intensities of Rhod-5N from a high calcium sample (red solid line) and low calcium sample (red dashed line); the fluorescence intensities of AF488 from a high calcium sample (green solid line) and low calcium sample (green dashed line); and corresponding Rhod-5N/AF488 ratios from the high calcium sample (black solid line) and the low calcium sample (black dashed line). **(B)** The Rhod-5N/AF488 ratios at different dye concentrations. The concentration of Rhod-5N and AF488 dye pairs at in vivo experiment condition is defined as 1. **(C)** Representative ratiometric image of Rhod-5N/AF488 and the clearance coefficient map of Rhod-5N and AF488, respectively. (n= 25 BM cavities, N= 10 mice) **(D)** Ratiometric image of interstitium and vessels right after dye injection (T1), 7 minutes after injection and no clearance correction (T2) and 7 minutes after injection with clearance correction (T2 after correction). The corresponding intravascular Rhod-5N/AF488 ratios at T1, T2 without clearance correction and T2 with clearance correction (n= 34 subregions from an intravascular space). Mean ± s.d.

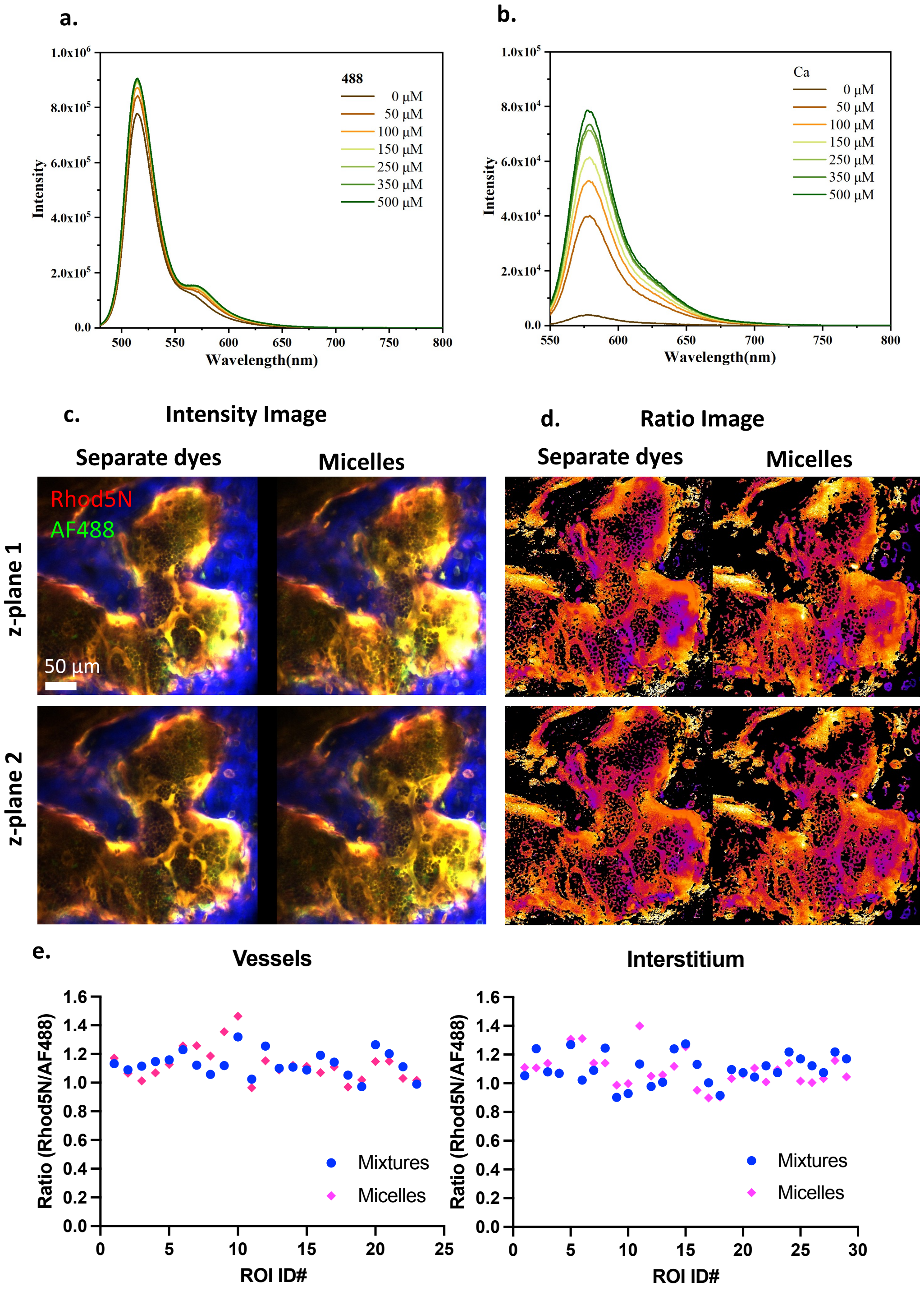

Supplementary Figure 7. Validation of Rhod5N/AF488 biodistribution using micelles. (**A-B**) The dye-loaded micelles are responsive to calcium in the Rhod5N channel while the AF488 intensity remained steady **(C-D)** Side by side comparisons of Rhod5N/AF488 intensity and ratio images of the same BM cavity from co-injected dyes or micelle-packaged dye mixtures**. (**N= 1 mouse) **(E)** Intravascular and interstitial ratios measured from micelles or co-injected dyes from the same subregions yielded consistent readings.

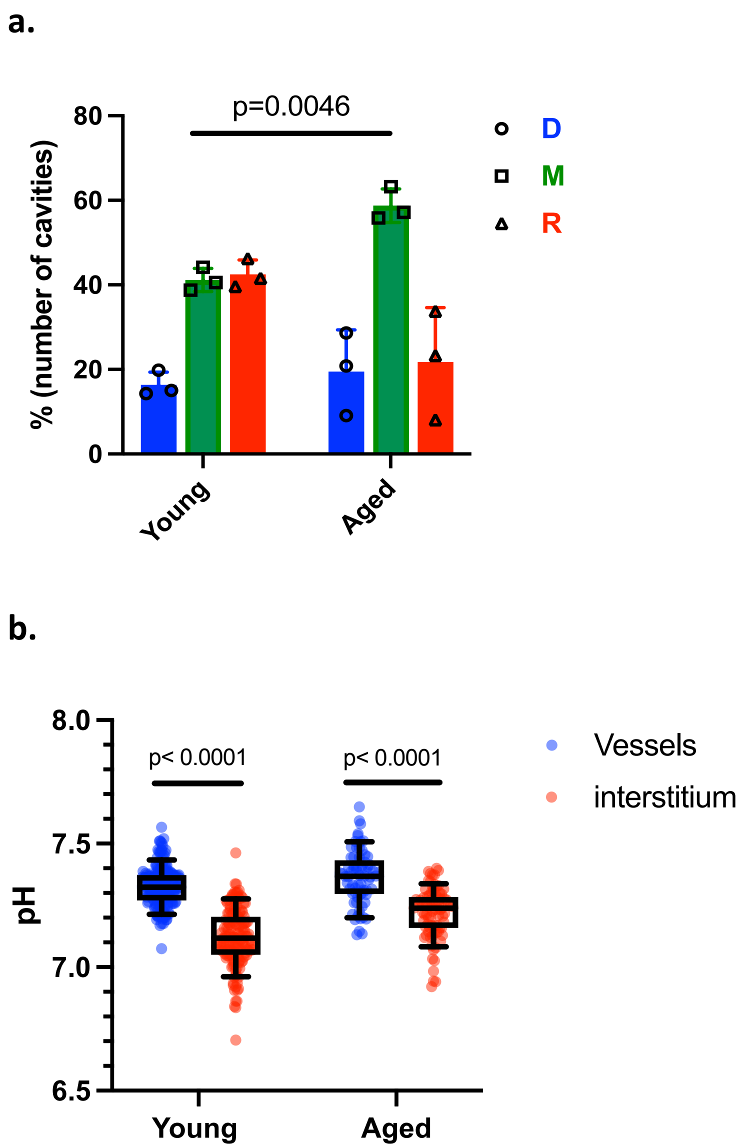

Supplementary Figure 8. Characterizations of bone remodeling and pH in aged animals. (A) M-type cavities increased significantly with age (N=3 animals per group, two-sided unpaired t-test. Mean ± s.d, p= 0.0046). **(B)** pH distribution in young and aged animals (N= 2 mice, n = 7 BM cavities. Two-sided Mann-Whitney test, p < 0.0001 between vessels and interstitial pH). Box and whiskers represent the median, 25 and 75 percentiles, and the 10-90% data range.

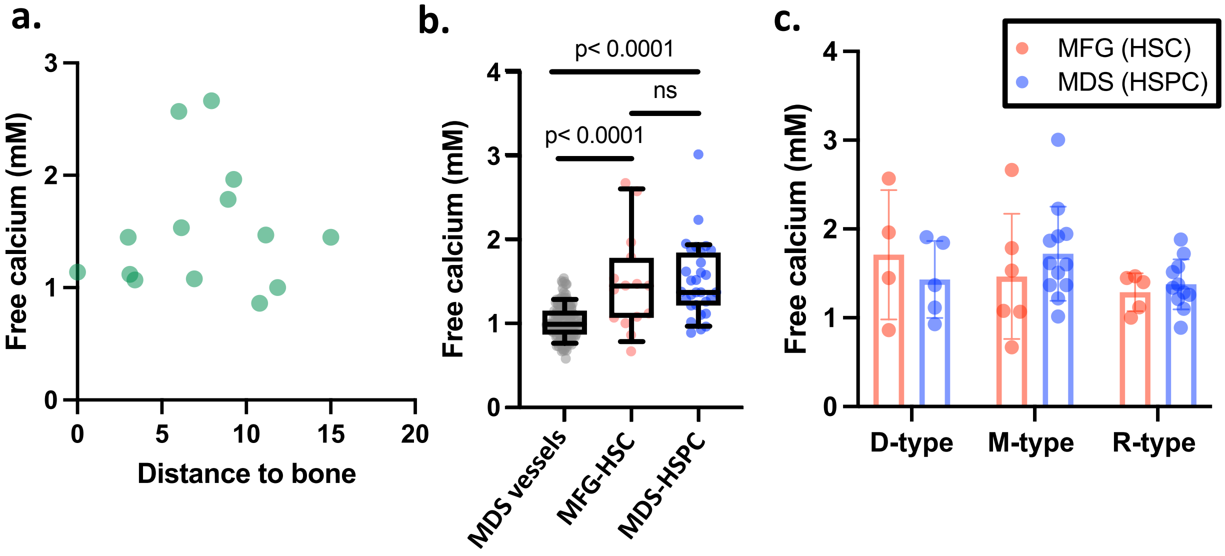

Supplementary Figure 9. Free calcium distribution near HSCs and HSPCs. (A) Measured $[Ca^{2+}]_e$ with respect to the distance to the endosteal surface from HSCs. **(B-C)** Free calcium measured in the vessels, and near long-term HSCs and HSPCs in different types of BM cavities. (N = 3 *MDS1*$^{GFP/+}$ mice, 7 BM cavities, n = 30 HSPCs; N= 5 *MDS1*$^{GFP/+}$*;FLT3*^{cre} (MFG) mice, 14 BM cavities, n = 15 MFG-HSCs). HSCs and HSPCs reside in locations with significantly elevated $[Ca^{2+}]_{e.}$ compared to serum calcium (Two-sided Mann–Whitney test. Mean ± s.d, p<0.0001). Box and whiskers represent the median, 25 and 75 percentiles, and the 10-90% data range.

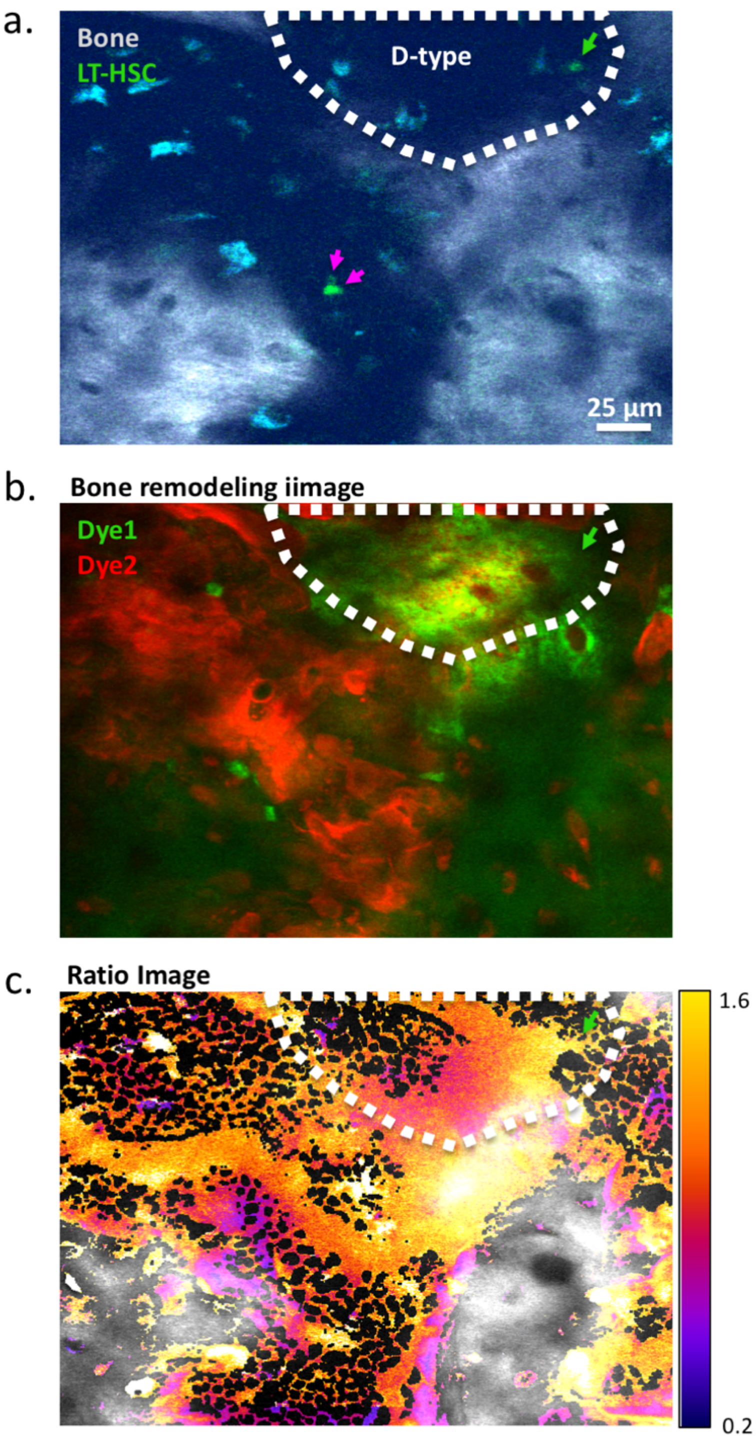

Supplementary Figure 10. LT-HSC in a D-type cavity was not found in low calcium regions. (A-B) A representative field of view and the corresponding bone remodeling staining showed *MDS1*$^{GFP/+}$*;FLT3*^{cre} LT-HSCs in a D-type (green arrow) and a R-type cavities (purple arrows). **(C)** Ratiometric imaging of Rhod-5N/AF488 revealed that the LT-HSC in the D-type cavity reside in a region with relatively high [Ca^{2+}] (green arrow). (n=4 D-type cavities, and n=5 R-type cavities)

a.

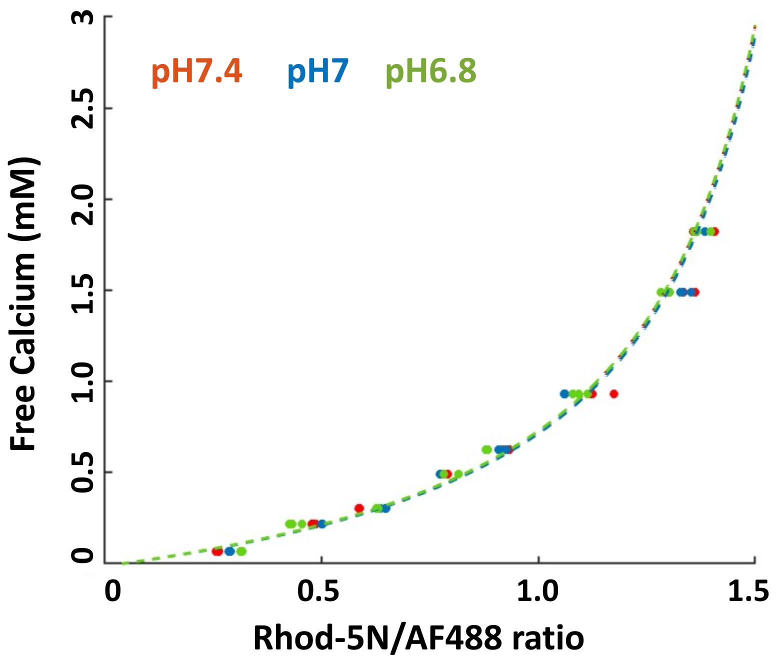

b.

	Keff	95 % CI	R squared
pH 7.4	0.6173	(0.6045- 0.6300)	0.9936
pH 7.0	0.6058	(0.5868-0.6248)	0.9850
pH 6.8	0.6165	(0.6067-0.6263)	0.9958

Supplementary Figure 11. Calcium calibrations curves (A) measured at pH 6.8 (green), pH 7.0 (blue) and pH 7.4 (red). **(B)** The K_{eff} = 0.62 (R^2 = 99.4%) at pH 7.4, K_{eff} = 0.61 (R^2 = 98.5%) at pH 7.0 and K_{eff} = 0.62 (R^2 = 99.6%) at pH = 6.8 (N=3 independent experiments).

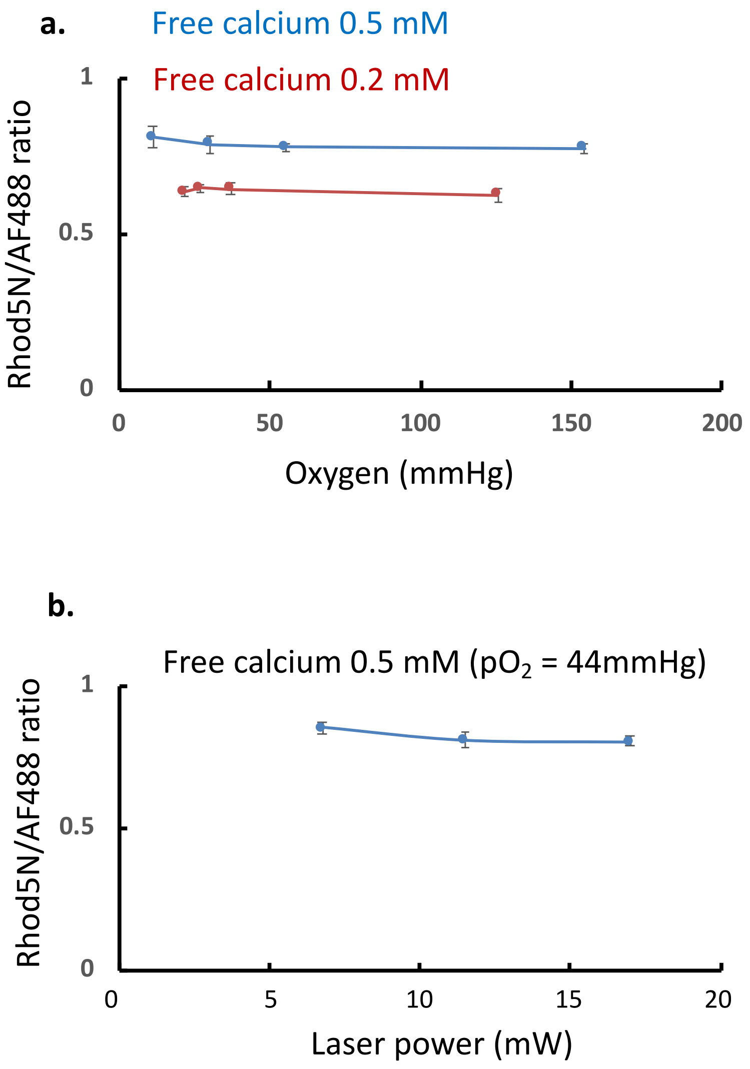

Supplementary Figure 12. Rhod5N/AF488 ratiometric measurements under hypoxia. (A) The Rhod5N/AF488 ratios with respect to pO_2 at two calcium concentrations (0.2 mM and 0.5 mM). (B) The Rhod5N/AF488 ratio as a function of laser power, measured at a $[Ca^{2+}]$ of 0.5 mM and pO_2 of 44 mmHg. Two-sided unpaired t-test. Mean ± s.d from 3 independent measurements.

a.

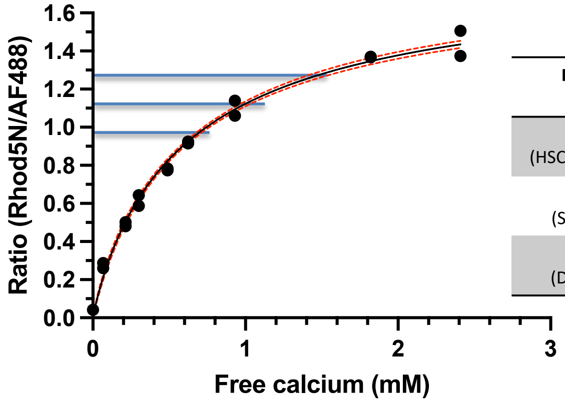

Ratio	Calcium (mean)	Uncertainty (90% CI)
1.27 (HSCs/HSPCs)	1.5 mM	± 0.06 mM
1.12 (Serum)	1.0 mM	± 0.03 mM
0.97 (D-type)	0.7 mM	± 0.02 mM

b.

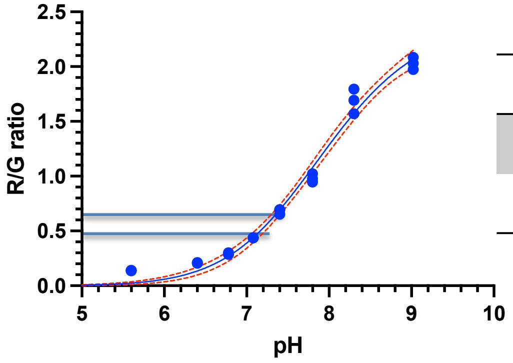

Ratio	pH (mean)	Uncertainty (90% CI)
0.63 (serum)	7.3	± 0.07
0.47 (Interstitium)	7.1	± 0.09

Supplementary Figure 13. The calcium and pH calibration curves fitted by the Hill equation. (A) The 90% confidence interval (red dashed lines) in the Hill slope shows larger uncertainty in the upper range of the calibration curve. The three horizontal lines indicate the average measured ratio from interstitial space surrounding HSCs/HSPCs, serum, and the D-type cavities. The table specifies the corresponding mean values and the uncertainty of calcium concentrations. (3 independent experiments) (B) The calibration curve for SNARF-1 including the 90% confidence interval (red dashed lines) in the Hill slope. The two horizontal lines indicate the average ratio from serum and interstitial pH. The table specifies the corresponding mean and the uncertainty of pH values. (3 independent experiments)

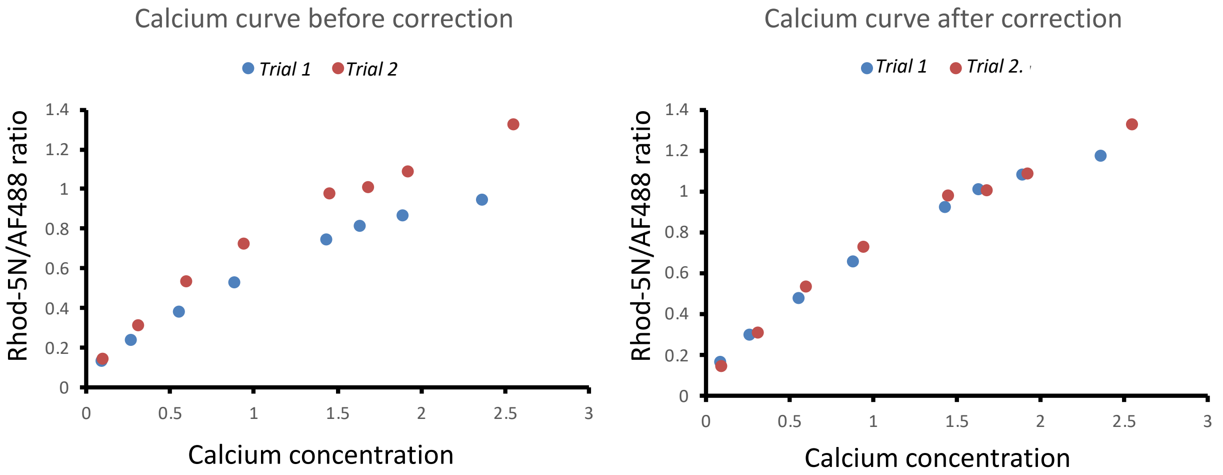

Supplementary Figure 14. **Calibration for ratio changes due to dye preparation. (A)** the raw calcium calibration curves generated by experiments on day1 (blue dots) and on day 2 (red dots). The Rhod-5N and AF488 were prepared and mixed independently. **(B)** the calcium calibration curves generated by experiments on day1 (blue dots) and on day 2 (red dots) with correction by equation 3.
