# Supplementary material for: Quantification of bone marrow interstitial pH and calcium concentration by intravital ratiometric imaging
Source: Nat Commun. 2022 Jan 19;13:393. doi: 10.1038/s41467-022-27973-x (PMC8770570; doi:10.1038/s41467-022-27973-x)
Supplement: Supplementary file 2 — Reporting Summary [file 41467_2022_27973_MOESM2_ESM.pdf]

nature research

Corresponding author(s): Charles P Lin

Last updated by author(s): Dec 18, 2021

Reporting Summary

Nature Research wishes to improve the reproducibility of the work that we publish. This form provides structure for consistency and transparency in reporting. For further information on Nature Research policies, see our Editorial Policies and the Editorial Policy Checklist.

Statistics

For all statistical analyses, confirm that the following items are present in the figure legend, table legend, main text, or Methods section.

n/a	Confirmed	
☐	☒	The exact sample size (*n*) for each experimental group/condition, given as a discrete number and unit of measurement
☐	☒	A statement on whether measurements were taken from distinct samples or whether the same sample was measured repeatedly
☐	☒	The statistical test(s) used AND whether they are one- or two-sided<br>*Only common tests should be described solely by name; describe more complex techniques in the Methods section.*
☒	☐	A description of all covariates tested
☒	☐	A description of any assumptions or corrections, such as tests of normality and adjustment for multiple comparisons
☐	☒	A full description of the statistical parameters including central tendency (e.g. means) or other basic estimates (e.g. regression coefficient) AND variation (e.g. standard deviation) or associated estimates of uncertainty (e.g. confidence intervals)
☐	☒	For null hypothesis testing, the test statistic (e.g. *F*, *t*, *r*) with confidence intervals, effect sizes, degrees of freedom and *P* value noted<br>*Give P values as exact values whenever suitable.*
☒	☐	For Bayesian analysis, information on the choice of priors and Markov chain Monte Carlo settings
☒	☐	For hierarchical and complex designs, identification of the appropriate level for tests and full reporting of outcomes
☒	☐	Estimates of effect sizes (e.g. Cohen's *d*, Pearson's *r*), indicating how they were calculated

Our web collection on statistics for biologists contains articles on many of the points above.

Software and code

Policy information about availability of computer code

Data collection	Calavaria imaging and in vitro imaging data collection was performed as described in detail in the methods section. The in vivo imaging procedures was also previously described in Spencer J.A. et al, Nature 2014 and Christodolou C. et al., Nature 2020. All analytical measurements (e.g. pH, pO2, Arsenazo assay) follow vendors' standard protocols and described in Methods.
Data analysis	Data analysis methodology for calvaria imaging and in vitro imaging is described in detail in the methods section.In addition, Graph Pad Prism (version 9) was used for the corresponding data analysis. For calvaria imaging data analysis, Matlab (R2019b), ImageJ scripts (v2.1.0/1.53c) were used. Several built-in plugins from image J were used, including contrast enhancement, background subtraction, and adaptive local thresholding. All Matlab codes and sample files are uploaded to: https://github.com/SCAYeh/ExtracellularCalciumAnalysis

For manuscripts utilizing custom algorithms or software that are central to the research but not yet described in published literature, software must be made available to editors and reviewers. We strongly encourage code deposition in a community repository (e.g. GitHub). See the Nature Research guidelines for submitting code & software for further information.

Data

Policy information about availability of data

All manuscripts must include a data availability statement. This statement should provide the following information, where applicable:

- Accession codes, unique identifiers, or web links for publicly available datasets
- A list of figures that have associated raw data
- A description of any restrictions on data availability

All data needed to evaluate the conclusions in the paper are present in the paper and the Supplementary Materials. Source data are provided with this paper. The

Matlab codes for image processing, the two-step depth correction, and clearance correction are available at: https://github.com/SCAYeh/ExtracellularCalciumAnalysis .

Field-specific reporting

Please select the one below that is the best fit for your research. If you are not sure, read the appropriate sections before making your selection.

- [x] Life sciences
- [ ] Behavioural & social sciences
- [ ] Ecological, evolutionary & environmental sciences

For a reference copy of the document with all sections, see nature.com/documents/nr-reporting-summary-flat.pdf

Life sciences study design

All studies must disclose on these points even when the disclosure is negative.

Sample size	No sample-size calculations were performed. The sample size was determined based on previous similar studies (Christodoulou et al. Nature, 2020) and was adequate based on consistency of measured results in each group.
Data exclusions	No data was excluded
Replication	Experimental findings were reliably reproduced. In rare cases in which large variability was observed it is indicated with corresponding SD. To verify reproducibility of the findings the vast majority of experiments were repeated three independent times.
Randomization	Weaned animals from Mds1-GFP/+ x Flt3-Cre crosses were separated in male and female cages. All of the double transgenic mice (Mds1-GFP/+; Flt3-cre) were males as Flt3-cre is carried by the y chromosome. The single transgenic Mds1-GFP/+ are females. Adult animals (4-6 months or > 70 weeks) of corresponding genotypes or wild type animals were then randomly selected from the cage and chosen for all experiments.
Blinding	Partial blinding was performed as data acquisition and analysis were mostly not performed by the same person. It is also not possible to determine the ratios during imaging so it was not possible to select any preferred regions for analysis. In all the calibration experiments, sample preparation and data acquisition were also not performed by the same person.

Reporting for specific materials, systems and methods

We require information from authors about some types of materials, experimental systems and methods used in many studies. Here, indicate whether each material, system or method listed is relevant to your study. If you are not sure if a list item applies to your research, read the appropriate section before selecting a response.

Materials & experimental systems

n/a	Involved in the study	
x		Antibodies
x		Eukaryotic cell lines
x		Palaeontology and archaeology
	x	Animals and other organisms
x		Human research participants
x		Clinical data
x		Dual use research of concern

Methods

n/a	Involved in the study	
x		ChIP-seq
x		Flow cytometry
x		MRI-based neuroimaging

Animals and other organisms

Policy information about studies involving animals; ARRIVE guidelines recommended for reporting animal research

Laboratory animals	All animals used in this study for BM and imaging analysis are of Mus musculus species, C57/BL6 background strain independent of genotype and 2-6 months of age or > 70 weeks (aged animals). Both males and females can be used for experiments. However, only male mice were used to visualize hematopoietic stem cells (Mds1-GFP/+ Flt3-Cre) because Flt3-cre is carried in the y chromosome. The mouse strain was generated as detailed in Christodoulou et al, Nature, 2020. All animals were housed in the virus-free barrier facilities or the satellite facilities, with a 12h light/ dark cycle and the temperature/humidity set to 68F/33%
Wild animals	The study did not involve wild animals.
Field-collected samples	The study did not involve field-collected samples.
Ethics oversight	Massachusetts General Hospital, IACUC protocol 2007N000148

Note that full information on the approval of the study protocol must also be provided in the manuscript.
