# Supplementary material for: Quantification of bone marrow interstitial pH and calcium concentration by intravital ratiometric imaging
Source: Nat Commun. 2022 Jan 19;13:393. doi: 10.1038/s41467-022-27973-x (PMC8770570; doi:10.1038/s41467-022-27973-x)
Supplement: Supplementary file 3 — Description of Additional Supplementary Files [file 41467_2022_27973_MOESM3_ESM.pdf]

Title: Supplementary Movie 1
Description: Intravital z-stack video (3 µm/z-step) starting from just above the endosteum into the bone marrow to show an example of pH imaging based on SNARF-1 dextran (Left) and the corresponding ratiometric illustration retrieved from SNARF1 R/G ratios (Right). SNARF1 (red, green), autofluorescent cells (green), bone (white, second harmonic generation). Scale bar ~100 µm. (n= 10 bone marrow cavities, N=2 mice).

Title: Supplementary Movie 2
Description: Intravital z-stack video (3 µm/z-step) starting from just above the endosteum into the bone marrow to show the comparison of ratiometric analyses before (Left) and after (Right) the depth correction to account for wavelength dependent signal attenuation from both bone and bone marrow. Note that autofluorescent cells is predominately in the green channel, and therefore exhibit low Red/Green ratio (blue patches in the interstitial space). Bone (white, second harmonic generation). Scale bar ~100 µm. (n= 10 bone marrow cavities, N=2 mice).

Title: Supplementary Movie 3
Description: Intravital z-stack video (3 µm/z-step) starting from the endosteum into the bone marrow to show an example of calcium imaging based on Rhod-5N (red) paired with AF488 (green). Autofluorescent cells are also visible in the green channel. Bone SHG signal is shown in white. Scale bar ~50 µm. (N=10 mice).

Title: Supplementary Movie 4
Description: Real time video (30 frame/second) of a single image plane in the bone marrow to show an instantaneous reduction of Rhod-5N signals (red) upon the administration of a calcium chelator, Calcein Blue. AF488 and autofluorescent cells both appear in the green channel; bone (SHG signal) is shown in white. Scale bar ~100 µm.

Title: Supplementary Movie 5
Description: Intravital z-stack video (3 µm/z-step) starting from just above the endosteum into the bone marrow to show the depth-corrected, ratiometric calcium imaging from Rhod-5N/AF488 (Left) and the corresponding bone remodeling status (M- and R-type) based on Calcein Blue and Alizarin Red (Right). Bone (white, second harmonic generation), Calcein Blue (blue), Alizarin Red (red). Scale bar ~50 µm. (N=10 mice).

Title: Supplementary Movie 6
Description: Intravital z-stack video (3 µm/z-step) starting from just above the endosteum into the bone marrow to show the depth-corrected, ratiometric calcium imaging from Rhod-5N/AF488 (Left) and the corresponding bone remodeling status (D-type) based on Calcein Blue and Alizarin Red (Right). Bone (white, second harmonic generation), Calcein Blue (blue), Alizarin Red (red). Scale bar ~50 µm. (N=10 mice).
